# Relation between bone mineral density and oxidative stress in Egyptian patients with chronic kidney disease: a cross sectional study

**DOI:** 10.1186/s12882-025-04099-y

**Published:** 2025-04-18

**Authors:** Samah M. Akab, Hanaa Elsayed Abozeid, Seham A. Elazab, Sherien Abdallh Fathy Elazab, Noran ElBazzar, Eman Refaat Youness, Mohamed Ahmed Shahba, Hisham A. Orban, Hanaa Reyad Abdallah, Moushira Zaki

**Affiliations:** 1 Department of Internal Medicine, Faculty of Medicine (Girls), Al-Azhar University, Cairo, Egypt; 2 Department of Rheumatology and Rehabilitation, Faculty of Medicine (Girls), Al-Azhar University, Cairo, Egypt; 3 Health Insurance, Nasr City, Egypt; 4 Department of Internal Medicine, Faculty of Medicine, Banha University, Banha, Egypt; 5 Department of Medical Biochemistry, Medical Research and Clinical Studies Institute, National Research Centre, Cairo, Egypt; 6 Biological Anthropology Department, Medical Research and Clinical Studies, Institute– National Research Centre, Cairo, Egypt

**Keywords:** Bone mineral density (BMD), Osteoporosis (OP), Chronic kidney disease (CKD), Oxidative stress (OS), Malondialdehyde (MDA), Paraoxonase-1 (PON-1), 8-hydroxy2-deoxy guanosine (8-OHdG)

## Abstract

**Background:**

Chronic kidney disease (CKD) patients are prone to osteoporosis (OP) and they had significant oxidative stress. The relationship between oxidative stress (OS) and bone mineral density (BMD) in CKD is not entirely clear. The investigation of this relation is of pronounced importance in decreasing the occurrence of osteoporosis among CKD cases.

**Objectives:**

To evaluate the association between BMD and OS in CKD patients.

**Methods:**

We performed a case-control study, including 150 adults with CKD (stage 1–5 according to Kidney Disease Improving Global Outcomes (KDIGO) classification, 2024) and 150 healthy controls. CKD patients were further subdivided to 3 subgroups based on estimated glomerular filtration rate: stage 1–2, stage 3–4 and stage 5. BMD at the lumbar spine (LS), femur neck (FN), and distal radius (DR) were measured using DEXA. Vitamin D and OS biomarkers including; 8-Hydroxy-2’-deoxyguanosine (8-OHdG) and Malondialdehyde (MDA) were measured. Paraoxonase1 (PON1) as a biomarker of antioxidant response was assessed. Statistical analysis was performed using the appropriate tests.

**Results:**

The CKD cases showed lower BMD T-Scores than healthy controls. Moreover, LS, DR, and FN BMDs were significantly different between CKD stages. Post hoc analyses showed higher LS, DR, and FN T-Scores in Stage I-II vs. Stage III-IV and Stage V. However, no significant differences were noted between stage III-IV and stage V for all sites. Significant increase in OS biomarkers (8-OHdG and MDA) while decreasing antioxidant activity (PON-1) with CKD severity were observed. There was a significant positive correlation between PON1and BMD while 8-OHdG and MDA had a negative correlation with BMD. We also observed significant positive correlations between 8-OHdG and MDA with alkaline phosphatase and phosphorus, while these markers had significant negative correlations with vitamin D and calcium. PON1 had a significantly positive correlation with vitamin D & calcium.

**Conclusion:**

CKD patients suffer of OS. OS positively correlated with CKD severity. There is a negative relation between OS and BMD in CKD. OS might participate in the occurrence of OP in CKD.

## Background

Chronic kidney disease (CKD) is a general worldwide health problem [1]. CKD is defined by KIDIGO, 2024 as abnormalities of kidney structure or function, present for a minimum of 3 months, with implications for health. CKD is classified based on cause, glomerular filtration rate (GFR) category (G1–G5), and albuminuria category (A1–A3), abbreviated as CGA [2]. Bone fractures are common in patients with CKD [3].

CKD is associated with the development of mineral bone disorder (MBD), OP, and fragility fractures [4]. CKD-related OP is a major complication in patients with CKD, conferring a higher risk of adverse outcomes [5]. Fractures were reported to be greater than 2 to 100-fold more common in CKD than in age-matched individuals without CKD [6–8].

Data from the National Health And Nutrition Examination Survey (NHANES) suggested that CKD and OP are highly co-prevalent [7, 9]. Therefore, Moe suggested that the term CKD-induced OP [10]. OP was two-fold as present in cases having eGFR < 60 mL/min than cases having eGFR > 60 mL/min amongst the NHANES III contributors [7].

OP is a global disease characterized by reduction of bone mass and alteration of bone architecture resulting in increased bone fragility and increased fracture risk [11, 12]. According to the National Institutes of Health Consensus Development Panel on Osteoporosis, OP is defined as a skeletal disorder characterized by compromised bone strength leading to an increased risk of fracture [13].

Moreover, according to the World Health Organization (WHO) criteria, OP is defined on the basis of T-score which is BMD ≥ 2.5 SDs below young adult mean. This definition of OP is for postmenopausal women and men above 50 years. It has to be mentioned that WHO criteria must not be used for men below 50 years, pediatric population, or premenopausal females. For those individuals, the International Society for Clinical Densitometry (ISCD) endorses applying the Z score (age and sex norms). Z scores ≤ − 2.0 are below the expected range for age and a secondary causes of OP should be searched for [14].

OS is the disturbance between oxidants and antioxidants towards increase in oxidants, causing a disturbance of redox signaling and/or injury of the molecules [15]. It is well-recognized that OS participates in CKD pathogenesis. It has been linked to OP also [16]. Disturbances in cell management of oxidants affects renal cell signaling, endorsing apoptosis and senility of kidney cells and reducing the capability of cells to regenerate. Thus, affecting renal function deleteriously [17].

The primary endogenous systems involved in the synthesis of oxidants comprising reactive oxygen species (ROS) like hydrogen peroxide (H2O2) and superoxide anion (O^2•−^) during normal metabolism is NADPH oxidase. Efficient antioxidant systems, such as vitamins E and C, glutathione peroxidase, reduced glutathione, superoxide dismutase, paraoxonase, and catalase, often balance out this creation of ROS under healthy conditions [18].

When the intracellular production of ROS is controlled, they act as second messengers by regulating and activating signaling transduction pathways involved in numerous biological processes such as apoptosis, survival, differentiation, proliferation, and inflammation [19]. Bone repair and bone remodeling are also redox -regulated processes, and the physiological redox state is essential for the equilibrium between osteoblastogenesis and osteoclastogenesis [20].

OS impairs mitochondrial function in osteoblasts, reducing ALP expression, activity, and mineralization capacity [21]. It promotes osteoclasts formation and differentiation. Studies showed that ROS can directly or indirectly stimulate osteoclast differentiation, augmenting their activity and numbers. The transient production of ROS due to transformation of receptor activator of nuclear kB ligand (RANKL) into osteoclastic precursor is very important for its role in the induction of osteoclastogenesis, and this indicates that ROS act as intracellular mediators for osteoclastic differentiation. This is through RANKL activation of the differentiation and activity of osteoclasts by interacting with specific receptors in pre-ostoeclasts and mediates osteoclastogenesis and bone resorption [21]. Moreover, H_2_O_2_ increases TRAP expression and upregulates RANKL expression, promoting osteoclast differentiation and activity [22].

Thus, OS has the ability to change the equilibrium between osteobalstogenesis and osteoclastogenesis. OP results from this change because it reduces bone mass. Specifically, increased ROS generation decreases osteoblastogenesis and osteoblastic activity while increasing osteoclastogenesis [23].

However, conflicting results about the association between BMD and OS in CKD have been reported. We hypothesized that the OS state in CKD patients plays a role in the associated reduction of BMD. Consequently, we carried out our study to assess the relation between BMD and OS in CKD patients.

## Methods

### Sample size calculation

Setting the power = 0.80 and α = 0.05 with using PASS 11th release [24], a minimal sample size of 99 cases was required to get statistical significance between null value of correlation coefficient (0.000) and assumed correlation coefficient (0.278) between T-scores at the spine correlated with phosphate in cases with CKD [25]. We included 150 cases with CKD and equal number (150) of healthy control subjects.

### Ethical approval

The study was approved by the Ethical Committee of Al-Azhar University (No. 1758) according to the World Medical Association’s Declaration of Helsinki. Written informed consent was obtained from patients after explaining the study for them.

#### Type of the study, settings and participants

We conducted a case-control, cross sectional study, including 150 adults attending the Nephrology Clinic of Alzahraa University Hospital with CKD not receiving hemodialysis (stage 1–5 according to KDIGO, 2024 [2]) and 150 normal healthy volunteers, as controls. Controls had no complaints, history of chronic disease, or abnormal clinical examination findings. CKD patients were subdivided to three groups according to estimated glomerular filtration rate (eGFR); stage 1–2, stage 3–4 and stage 5, none of our patients was on hemodialysis.

Stage 1 CKD was defined as cases with eGFR ≥ 90 mL/min/1.73m^2^, stage 2 CKD as eGFR 60 to 89 mL/min/1.73m^2^, stage 3 CKD as eGFR 30 to 59 mL/min/1.73 m^2^, stage 4 as those with eGFR 15–29 mL/min/1.73m^2^, and stage 5 as those with eGFR < 15 mL/min/1.73m^2^. For eGFR calculation, we adopted the Chronic Kidney Disease Epidemiology Collaboration equation (CKD-EPI) [26].

### Exclusion criteria

- Pregnant, breastfeeding females as hormonal changes can significantly affect bone metabolism.
- Individuals with excessive alcohol consumption as well as tobacco smokers were excluded from the study.
- Patients with acute inflammation, active infection, malignant diseases, metabolic, congenital bone disease or previous lumbar vertebrae surgery, patients who are frequently exposed to ionizing radiation or regularly use drugs lead to osteoporosis like corticosteroids or those regularly use drugs treating osteoporosis (e.g. teriparatide, bisphosphonates & estrogen).

### Procedures

#### I-Clinical evaluation

The following was done for all included individuals:

- History taking and thorough clinical examination.
- Anthropometric assessment including; body weight to the nearest 0.1 kg and height to the nearest 0.1 cm were taken according to the instructions of International Biological Program [27]. Body mass index (BMI) was calculated according to the equation; weight (Kg) / height (meter^2^).

#### II-Evaluation of BMD

For OP identification, dual-energy X-ray absorptiometry (DEXA) is the corner stone. The principle of its action is as follows; when X-rays move throughout bodily tissues, their strength is weakened to varying degrees inside various types of tissues. It uses X-rays at two energy levels. The best beneficial sites are the lumbar vertebrae and neck of femur [28]. In the current study we measured BMD at three sites; femoral neck, lumbar spine and distal radius as they represent the BMD for the bones of the body in general and not only specific bones. Clinically, BMD is usually used to diagnose OP. Cancellous bone (60–75%) in the total skeletal system is concentrated in the lumbar spine, thus the initial alterations in bone mineral content can be reflected by assessment of lumbar BMD in CKD cases. Moreover, the rate of bone turnover in cancellous bone is greater than that of cortical bone and it is more sensitive to metabolic stimuli. Additionally, its accumulation of bone mass will reach the topmost before the cortical bone, hence the assessment of lateral lumbar BMD is having greater sensitivity [29].

In addition, the femoral neck, lumbar spine and distal radius are part of the standardized protocol recommended by the WHO and the International Society for Clinical Densitometry (ISCD) to measure the BMD of the body bones. These sites are chosen because they are the most predictive of fracture risk and are widely accepted in clinical practice world-wide [30]. DEXA was done using GE Healthcare Care lunar IDXA with central scan device.

### a-Appropriate patient positioning

It is significant for the patient to be put in the right position to get a better evaluation of BMD for diagnosing OP. During lumbar spine and femoral neck measurement, the patient should be placed in a supine position and when it comes for imaging of the forearm he should sit next to the table. In proximal femur scans requires the patient to rotate the foot internally so as the femoral neck will be parallel to the table and the lesser trochanter will be hidden as much as he can. improper patient positioning can cause under- or over-estimation of BMD [31].

### b-BMD interpretation

BMD should be expressed as raw values (g/cm^2^) as well as T scores. However, in clinical practice, raw BMD values (g/cm^2^) are not normally used for assessing skeletal status and fracture risk. Instead, the BMD values are expressed in terms of the number of standard deviations above or below the young normal value (commonly referred to as the T-score) [32].

According to the WHO, OP was diagnosed in cases with BMD T score less than or equal to − 2.5, while osteopenia was diagnosed with T score between − 1 and − 2.5 and normal BMD was considered if T score higher than − 1 [33].

#### III-Laboratory investigations

5 ml venous blood were taken while fasting, and put in plain tubes, left to clot then centrifuged and serum was separated and kept at -80 C till analysed to assess:

1. Serum creatinine, albumin, calcium, phosphorous, and alkaline phosphatase using the Hitachi 704 Auto-analyzer and colorimetric enzymatic techniques (Roche Diagnostics. Switzerland).
2. Measurement of OS biomarkers:
  - Lipid peroxidation (MDA) assay: Using Nair and Turne’s approach [34], Malondialdehyde (MDA), a byproduct of lipid peroxidation, was measured in serum. That assessment produces the TBA-MDA adduct, that could be detected colorimetrically at 532 nm, when thiobarbituric acid and thiobarbituric acid reactive substances (TBARS) react.
  - Estimation of 8-Hydroxy-2’-deoxyguanosine (8-OHdG) serum levels: Using ELISA kits, serum concentrations of 8-OHdG were measured twice according to Sinogene Clon Biotech Co. Catalog No. SL- 2044 Hu.
3. Assessment of serum Paraoxonase-1 (PON-1) as an antioxidant: Phenyl acetate was used as a substrate in a colorimetric technique to measure PON-1’s arylesterase activity. PON-1 catalyses the cleavage of phenyl acetate, which forms phenol. The increase in absorbance at 270 nm at 25 °C was used to calculate the rate at which phenol was forming. The working mixture included 4 mM phenyl acetate as the substrate and 20 mM Tris/HCl buffer, pH 8.0, with 1 mM CaCl2. After adding samples diluted 1:3 in buffer to the mixture above, a 20-second lag time was used to record the changes in absorbance. One µmole of phenol generated per minute is equivalent to one unit of arylesterase activity. Based on phenol’s 1310 M‒1 cm‒1 extinction coefficient, PON1 activity was represented in kU/L. Water-containing blank samples were utilized to account for phenyl acetate’s spontaneous hydrolysis [35].
4. Estimation of 25- OH vit D3 serum levels: Vitamin D direct ELISA Kit (EIA-4696) (DRG ^®^ International, Inc. USA) was used to measure serum 25 hydroxy vitamin D (25 (OH) vit D3).
5. Estimation of PTH levels: PTH was measured using the enzyme-linked immune-sorbent assay (ELISA) in line with ELK Biotech Co. Catalog No. ELK2427.

### IV-Statistical analysis

Data were analyzed using SPSS version 20.0 (Statistical SPSS Inc, Chicago, IL, USA). The normality of the data was examined using the Shapiro-Wilk test. Non-parametric statistics were demonstrated as median (minimum-maximum) and IQR, but parametric variables were demonstrated as mean ± SD. Participant characteristics were first compared between CKD and the control group using Student’s t-test, for normally distributed data while Mann-Whitney U test was used for non-parametric data. One-way analysis of variance (ANOVA) was used to compare between means of the studied subgroups at different CKD stages followed by independent-sample t-test for subgroups with normally distributed data using post-hoc analysis while Kruskal-Wallis test was used for non-parametric data followed by Mann- Whitney U test. Qualitative data were described as number and percentage and compared using Chi square test and Fisher’s Exact test for variables with small expected numbers. Correlation between BMD and OS biomarkers was done using Pearson correlation coefficient test. Multiple regression analysis of both BMD and OS variables was assessed. P-values below 0.05 were considered significant in all analyses.

## Results

Table 1 shows comparison of demographic data and anthropometric measures between the controls and total CKD patients (P1). The different stages of CKD were compared together (P2). Neither age, weight, sex distribution nor BMI differed significantly (*p* > 0.05) between the total CKD patients and the controls or between the three subgroups of CKD.

**Table 1:** Demographic data and anthropometric measurements of the total CKD patients and subgroups

Measure	Control *N* = 150	Total studied CKD patients *N* = 150 (100%)	P1	Stage I-II *N* = 45 (30%)	Stage III-IV *N* = 50 (33.33%)	Stage V *N* = 55 (36.67%)	P2
Age (year)	49.00 ± 10.74	56.29 ± 15.20	0.233	50.40 ± 6.24	59.80 ± 12.02	58.45 ± 10.71	0.223
Sex Males Females Total	86 (57.33%) 64 (42.67%) 150 (100%)	90 (60%) 60 (40%) 150 (100%)	0.421	26 (57.78%) 19 (42.22%) 45 (100%)	29 (58%) 21 (42%) 50 (100%)	32 (58.18%) 23 (41.82%) 55 (100%)	0.323
Weight (Wt) (kg)	79.80 ± 9.067	78.61 ± 20.69	0.424	83.70 ± 20.17	72.1 ± 10.32	79.91 ± 27.43	0.56
Height (Ht) (m)	1.67 ± 0.04	1.63 ± 0.63	0.05	1.63 ± 0.08	1.62 ± 0.06	1.64 ± 0.06	0.011
Body Mass Index (kg/m^2^)	27.09 ± 2.74	29.01 ± 6.63	0.334	31.55 ± 7.41	27.94 ± 4.67	27.54 ± 7.35	0.321

SD; standard deviation, BMI; Body Mass Index

P1 = significance of students t-test between total cases and control, P2 = significance of ANOVA test between subgroups

Regarding the clinical data of our cases, there were no significant difference between the studied groups, (*p* > 0.05) for all as shown in Table 2.

**Table 2:** Clinical data of the studied groups

Variable	Control *N* = 150	Total studied CKD patients (*n* = 150)	P1	Stage I-II *N* = 45	Stage III-IV *N* = 50	Stage V *N* = 55	P2
Fracture prevalence	0 (0%)	4 (2.7%)	0.07	0(0%)	1(2%)	3 (5.5%)	0.06
Menopaused femlaes	7 (4.7%)	8 (5.3%)	0.06	2 (4.4%)	3(6%)	3 (5.5%)	0.07
Use of vitamin D analouge	10 (6.7%)	32 (21.3%)	0.58	3 (6.7%)	5 (10%)	24 (43.6%)	0.06
Use of phosphate binder	0 (0%)	15 (10%)	0.67	0 (0%)	5 (10%)	10 (18.2%)	0.57

Chi square test; *p* < 0.05 = significant

Table 3 shows comparison of BMD T-scores among the studied groups. Total CKD patients had lower T-scores for LS, DR and FN compared to healthy control group (*p* = 0.001), whereas cases with StageI-II had higher T-scores compared to Stage III-IV and Stage V (*p* < 0.05), patients with advanced CKD had lower T-scores based on ANOVA and post hoc test. While no significant difference was found for all T-scores between Stage III-IV and Stage V (*p* > 0.05).

**Table 3:** BMD T-scores among the studied groups

T-score	Control *N* = 150	Total studied CKD patients *N* = 150	P1	Stage I-II *N* = 45	Stage III-IV *N* = 50	Stage V *N* = 55	P2
LS	0.88 ± 0.33	-1.52 ± 0.10	0.001	0.63 ± 0.37^^^	-1.64 ± 0.09	-1.32 ± 0.11	0.001
DR	0.80 ± 0.26	-1.45 ± 0.05	0.001	0.18 ± 0.34^^^	-1.75 ± 0.06	-1.33 ± 0.52	0.001
FN	0.86 ± 0.22	-1.61 ± 0.32	0.001	0.06 ± 0.97^^^	-1.89 ± 0.72	-1.85 ± 0.80	0.001

LS: lumbar spine, DR: Distal radius, FN: Femur neck.P1 = significance of student‘s t-test between total cases and control, P2 = significance of ANOVA test between subgroups, post hoc analysis: *p* < 0.01 Stage 1–2 vs. Stage 3–4 and Stage 5

Table 4 shows a significant increase of creatinine, phosphorus, alkaline phosphatase, 8-OHdG, MDA, and PTH in total CKD patients compared to the control, while Vitamin D, Ca, albumin, and PON1 decreased. Regarding the values in these markers among the patients with different CKD stages, there were significant differences (*p* < 0.05), post hoc analysis revealed statistically significant difference in all the studied laboratory biomarkers among patients with stage I-II vs. cases with stage III-IV and stage V CKD (*p* < 0.05).

**Table 4:** Laboratory findings among the studied groups

Marker	Control *N* = 150	Total studied patients (*N* = 150)	P1	Stage I-II *N* = 45	Stage III-IV *N* = 50	Stage V *N* = 55	P2
Creat(mg/dl) (M: 0.7–1.3, F: 0.6–1.1) mg/dl	0.86 ± 0.17	4.25 ± 1.21	0.01	1.69 ± 0.42	2.49 ± 0.44	5.36 ± 1.37	0.001
Ca (mg/dl) (8.5–10.2) mg/dl	9.76 ± 0.62	8.37 ± 1.18	0.01	9.73 ± 0.54	9.00 ± 0.71	8.25 ± 1.29	0.001
Phos (mg/dl) (2.5–4.5) mg/dl	3.32 ± 0.47	4.21 ± 0.84	0.01	3.95 ± 0.73	4.28 ± 0.93	4.60 ± 1.09	0.01
PTH (pg/ml) (15–65) pg/ml	51.40 ± 6.16	180.39 ± 13.55	0.001	48.90 ± 7.75	165.50 ± 3.55	341.64 ± 21.66	0.001
Alb (g/dl) (3.4–5.4) g/dl	3.79 ± 0.16	3.33 ± 0.41	0.001	3.57 ± 0.34	3.35 ± 0.65	3.28 ± 0.45	0.05
Vit D(ng/ml) (20–40) ng/ml	28.30 ± 6.97	13.77 ± 6.32	0.001	19.80 ± 4.71	13.10 ± 5.02	8.91 ± 3.80	0.001
ALP (IU/L) (44–147) IU/L	55.40 ± 1.70	139.90 ± 9.18	0.001	62.80 ± 13.16	128.90 ± 8.08	143.82 ± 10.23	0.001
8-OHdG (pg/ml)	1.86 ± 7.60	5.78 ± 16.10	0.001	2.58 ± 09.19	3.78 ± 06.0	6.63 ± 07.01	0.001
MDA (nmol/l)	2.37 ± 0.41	6.42± 1.27	0.001	4.58 ± 2.18	6.61 ± 1.31	7.91 ± 1.94	0.001
PON-1 (U/L)	116.8 ± 9.22	50.59 ± 15.80	0.001	95.78 ± 7.13	51.45 ± 0.14	36.91 ± 16.50	0.001

Creat: Creatinine, Cal: Calcium, Phos: Phosphorus, Alb: Albumin, Vit D: Vitamin D, ALP: Alkaline Phosphatase, 8OHdG: 8-Hydroxy-2’-deoxyguanosine, MDA: Malondialdehyde, PON-1: Paraoxonase-1, PTH: parathyroid hormone. P1 = significance by Student’s t test between total cases and control, P2 = ANOVA between subgroups. *p* < 0.05 by post hoc analysis, Stage 1–2 vs. Stage 3–4 and Stage 5

Our results revealed also a significant positive correlation between both MDA and 8-HdOG with alkaline phosphatase and phosphorus. However, they had a significant negative correlation with vitamin D and calcium. PON-1 was negatively correlated significantly with alkaline phosphatase, PTH & phosphorus, whereas, it positively correlated significantly with vitamin D and calcium. Moreover, there was a significant positive correlation between PON1and BMD as measured by T-scores of LS, DR and FN, meanwhile, 8-OHdG and MDA had a negative correlation with BMD as shown in Table 5.

**Table 5:** Correlation of OS biomarkers with BMD and biochemical findings in total CKD patients

Variable	MDA *R*	PON1 *R*	8-OHdG *R*
MDA	1	− 0.873^**^	0.826^**^
PON 1	− 0.873^**^	1	− 0.714^**^
ALP	0.785^**^	− 0.690^**^	0.752^**^
8-OHdG	0.826^**^	− 0.714^**^	1
Ca	− 0.539^**^	0.553^**^	− 0.524^**^
Phos	0.448^**^	− 0.396^*^	0.429^**^
PTH	0.284	− 0.349^*^	0.282
Vit D	− 0.782^**^	0.694^**^	− 0.723^**^
LS	− 0.443^**^	0.391^*^	− 0.290
DR	− 0.689^**^	0.547^**^	− 0.617^**^
FN	− 0.711^**^	0.536^**^	− 0.649^**^

Pearson correlation Coefficient test; ** Correlation is significant at the 0.01 level (2-tailed). LS: lumbar spine, DR: Distal radius, FN: Femur neck

Table 6 shows stepwise multiple regression analysis of BMD T-scores of LS, FN and DR with OS in CKD patients. MDA, PON1, and 8-OHdG were included as independent variables while BMD T-scores as dependent variable. Significant association between each of MDA, 8-HdOG, and PON1 with T-scores was observed.

**Table 6:** Stepwise multiple regression analysis of BMD T-scores with OS markers in CKD patients

Dependent variable of LS BMD	*r* ^2^	Β	*P*
MDA	− 0.42	− 0.45	0.01
PON1	0.61	0.49	0.01
8-OHdG	− 0.89	− 0.51	0.01

Dependent variable of FN BMD	r^2^	Β	*P*
MDA	− 0.52	− 0.55	0.01
PON1	0.51	0.46	0.01
8-OHdG	− 0.88	− 0.52	0.01

Dependent variable of DR BMD	r^2^	Β	*P*
MDA	− 0.42	− 0.55	0.01
PON1	0.41	0.41	0.01
8-OHdG	− 0.81	− 0.42	0.01

MDA, PON1 and 8-OHdG were included as independent variables and BMD of LS, FN and DR as dependent variables

## Discussion

OS and acceleration of the inflammatory state are closely linked to the severity and course of CKD. Numerous investigations have documented decreased anti-oxidative systems and increased levels of pro-inflammatory enzymes, cytokines and OS indicators [36].

Several researches have proved that OS harmfully affects the process of bone remodeling, leading to decrease in BMD [37, 38]. Current documents showed that inflammation caused by OS plays a role in the etiology of OP [39]. Therefore, in this study; we intended to evaluate the association of BMD with OS in CKD.

In the current study, CKD patients with Stage1-2, Stage 3–4 and Stage 5 had lower T-scores for LS, DR and FN than the control group. Whereas, cases with Stage1-2 had higher T-scores as compared to Stage 3–4 and Stage 5. So, BMD had a significant negative association with the stage of CKD, which is coherent with prior researches that reported low BMD among stage 3–5 patients [40, 41].

Park et al., declared that reduction of BMD was prevalent in patients with end stage renal disease (Stage 5) and it was precipitating element to fracture; causing long-lasting inability in those patients [42]. This is in agreement with our results.

In end stage renal disease (ESRD) patients; excretion of phosphorus, diminished metabolism of vitamin D3, reduced calcium level, elevated PTH, chronic acidosis, old age, increased presence of diabetes as a causative factor and impaired nutritional intake are proposed to be reasons of bone illness [43, 44].

In the present study, we also observed a significant increase in alkaline phosphatase and PTH in CKD cases compared to the control group, while vitamin D levels were significantly decreased.

Agreeing with our results, Nickolas et al., proved that high levels of PTH were associated with low BMD [45]. Additionally, the association of this hormone with fracture was described mostly in patients on dialysis [46].

Moreover, it is a fact that elevated level of PTH expects damage of cortical area, density, thickness, and intensifies the porosity of cortical area, whereas it reduces the strength of bone, that creates OP [47]. Generally, increased 25(OH)D levels have a great effect on bone strength due to its active metabolite [1,25(OH)_2_D] action in intestines, parathyroid gland and bone [48, 49]. At clinical level, intake of precursors/analogues of vitamin D; ergocalciferol, cholecalciferol, and calcifediol were accompanied with decrease in PTH concentrations in CKD cases [50]. Therefore, administration of vitamin D significantly affects metabolism of bone and proper concentrations of vitamin D increases the effectiveness of anti-resorptive treatments, like; bisphosphonates [51].

Regarding the OS biomarkers in our study, we assessed MDA which is produced through lipid peroxidation and during prostaglandin and thromboxane synthesis and could attack big molecules, resulting in changes in their roles [52]. In many researches, greater serum MDA concentrations were shown in CKD cases than healthy controls [53, 54]. MDA differed significantly in CKD cases in stages 2, 3, 4, and 5 [55]. Higher levels of serum MDA were also found in haemodialysis patients [56]. Furthermore, serum MDA decreased after kidney transplantation [57].

The above mentioned findings coincide with the results of our study as we found significant increase in MDA in total studied cases with CKD compared to the controls (*p* = 0.001). It is worth noting that MDA levels differed significantly according to the severity of CKD (*p* = 0.001) in the current study.

This agrees with the cross-sectional study done by Sagar et al., on four hundred individuals including three hundreds cases having CKD and one hundred normal subjects as a control. MDA levels were significantly different between groups. Stage V cases had the highest levels compared to the other groups [58].

8-hydroxy-2-deoxyguanosine (8-OHdG), one of the greatest profuse products of DNA oxidation, is a sensitive OS biomarker which reflects very small levels of oxidative DNA destruction [59]. High concentration of serum 8-OHdG acts as a surrogate biomarker of oxidative DNA damage in dialysis cases [60, 61]. An elevated level of 8-OHdG in leukocyte DNA was detected in peritoneal and haemodialysis cases. They had the highest 8-OHdG level, then un-dialyzed CKD patients and healthy controls [61].

In this regards, we assessed 8-OHdG in our CKD patients and detected that the above findings coincide with the results of our study as we found significant increase in serum 8-OHdG in total studied cases with CKD compared to the controls (*p* = 0.001). Furthermore, in our study, we noticed an association of 8-OHdG with the severity of CKD.

Concomitant with our results, Dai et al., found that Serum 8-hydroxydeoxyguanosine (8-OHdG) increased with worsening of kidney function [62].

From another aspect, the findings of the present work are parallel to that of a meta-analysis done by Watanabe et al., on twenty four researches and found that CKD individuals had a lesser level of PON-1 than the control; proving that the antioxidant function of PON1 can be reduced in CKD [63].

Furthermore, in our study, we noticed a decrease of antioxidant biomarker (PON-1) with a further decrease in renal function.

On evaluating PON1 activity as a prognostic and diagnostic tool in CKD, Samouilidou et al., noticed that PON1 activity may be utilized in the assessment of antioxidant status in both dialyzed and non-dialyzed patients, the prediction of CVD in patients undergoing dialysis and transplantation, the assessment of the antioxidant effect of statins and nutritional supplementation, the assessment of nephropathy occurrence in individuals with T2DM, and the prognosis of future adverse clinical outcomes [64].

It was found that redox regulation of bone remodeling acts at the molecular level. A lot of information showed that higher ROS levels were connected with an intensification in apoptosis of osteocytes and/or osteoblasts, accountable for the damaged balance of bone remodeling. Especially, at the molecular level, in vitro researches proved that elevated ROS levels linked to osteocyte apoptosis had mitochondrial basis, and that c-Jun-N terminal kinase (JNK) activation through mitochondrial pathways is the principal action [65, 66]. OS-stimulated apoptosis is the result of the activation of caspase-3, -6, and − 7, initiated by the production of cytochrome C through the external mitochondrial membrane, to increase its permeability [67].

Thiol antioxidants, like GSH, lipoic acid (LA), and N-acetylcysteine (NAC; a cysteine analogue drug with therapeutic uses), can decrease ROS release and apoptosis due to caspase-3 activation; especially, apoptosis is prohibited by the specific prevention of JNK stimulation [65]. Antioxidants can also prevent ROS increase, JNK activation, and osteocyte apoptosis via controlled redox and non-redox mechanisms [65, 66, 68].

On the other hand, MDA has a powerful toxic effect on the cells, polymerizing macromolecules (proteins, lipids and DNA & RNA), strongly reflecting the speed and severity of lipid peroxidation. It could be considered as an indirect indicator for intensity of injury caused by free radicals, representing the procedure of ROS injury. In addition, MDA is able to prevent the protein and nucleic acid formation and reduce the enzyme action, leading to reduced antioxidants in the body and so progressively decreasing the bone mass. After OS induces the osteoclast growth and differentiation, osteoclasts will yield additional ROS, aggravate the impairment of the antioxidant defense system in the body and lead to a vicious circle, eventually causing severe OP [69].

A study done by Ren et al., investigated the relationship between OS and OP among ESRD patients on maintenance hemodialysis and found significantly greater MDA levels in those patients than the controls, demonstrating that maintenance hemodialysis cases having OP suffered a state of evident OS [70]. Ren et al., concluded also that MDA levels were negatively correlated with BMD and Hemodialysis CRF patients were prone to OP, having a significant OS status; its degree was closely related to OP [70]. This is compatible with our results.

In contrast to the results of the current study, Kayabaşi et al., reported that TAC significantly correlated positively with T score of FN only, while they couldn’t find any significant correlation between other studied OS biomarkers and T-scores of FN or LS in ESRD patients. They detected high OS and reduced antioxidant capacity. However, they couldn’t detect that OS had any impact of on OP in ESRD patients [71]. This disagrees with our results.

The role of OS in pathogenesis of OP can be attributed also to the occurrence of oxidants-antioxidants imbalance that can prevent the maturation of osteoblast precursors to osteoblasts, preventing osteoblasts mineralization then enhancing their demise. When OS induces the osteoclasts to grow and get into maturation, osteoclasts subsequently form extra ROS, aggravating the destruction of the antioxidant defending process inside the body. This produces a vicious circle and eventually causes severe OP [69].

Our study has a major strength that we assessed the relation between OS and BMD in all stages of CKD while up to our knowledge; all the previuos studies investigated the relation of OS and OP in ESRD (Dialyzed patients) only.

### Limitations of the study

Our study has some limitations; includig:

Firstly; the cross sectional design of our study which prevented us from follow up of patients with CKD until they develop the OP with regular measurement of OS biomarkers and administration of antioxidants to assess their effects in improving CKD and BMD and prevention of OP. Hence, further longitudinal follow up studies and clinical trials with greater number of patients are required to approve the results of the current study and endorse the usage of those OS biomarkers as indicators of CKD prognosis. Furthermore, prospective researches targeting possible dietary interferences to reduce OS and bone resorption in individuals with chronic renal disease are very essential.

Secondly, we measured only three biomarkers of OS which is not enough in assessment of OS status as the evaluation comprises a battery of investigations including; oxidants, antioxidant enzymes, elements and vitamins but we couldn’t do this as it would cost us too much and our study didn’t receive any fund; it was self funded.

Thirdly, the current research did not intervene with or assess the lifestyle effects in CKD individuals (nutritional status, dietary habits and physical activity) and additional risk factors which also contribute to OS and decrease BMD.

Fourthly, we didn’t investigate the CKD patients according to the cause of CKD and its relation to OS and BMD.

### Conclusions

The present study demonstrates that patients with CKD have low BMD being the lowest in stages 3–4 and 5, thus more predisposed to OP with a negative association between BMD and CKD severity. CKD patients suffer of OS. There is a negative association between OS and BMD. OS might participate in the occurrence of OP in CKD.

## Data Availability

The study Data are available upon request from the corresponding author.

## Abbreviations

Alp: Alkaline phosphate
aOR: adjusted odd ratio
BMD: Bone mineral density
Ca: Calcium
CKD: Chronic kidney disease
DEXA: Dual energy x-ray absorptiometry
DR: Distal radius
e-GFR: estimated glomerular filtration rate
ESRD: End stage renal disease
FN: Femur neck
GFR: Glomerular filtration rate
KIDGO: Kidney Disease Improving Global Outcomes
LS: Lumbar spine
MBD: Mineral bone disorder
MDA: Malondialdehyde
NHANES: National Health and Nutrition Examination Survey
OHdG: 8-Hydroxy-2’-deoxyguanosine
OP: Osteoporosis
OPG: Osteoprotegerin
OS: Oxidative stress
PTH: Parathyroid hormones
PON1: Paraoxonase1
RANKLE: Of receptor activator of nuclear factor-κb ligand
ROS: Reactive oxidative species
SMD: Standard mean difference
SOD: Superoxide dismutase
TAC: Total antioxidant capacity
TBARS: Thiobarbituric acid reactive substances
